# Association with CYP2C19 polymorphisms and Clopidogrel in treatment of elderly stroke patients

**DOI:** 10.1186/s12883-021-02127-6

**Published:** 2021-03-09

**Authors:** Changqing Li, Weihua Jia, Jian Li, Fangfei Li, Jing Ma, Lichun Zhou

**Affiliations:** 1 Department of Neurology, Beijing Chaoyang Hospital, Capital Medical University, Beijing, China; 2 Department of Neurology, Shijingshan Teaching Hospital of Capital Medical University, Beijing Shijingshan Hospital, Beijing, China; 3 Department of Echocardiography, Shanghai Xuhui Central Hospital, Zhongshan-Xuhui Hospital, Fudan University, Shanghai, China

**Keywords:** CYP2C19, Clopidogrel, Stroke, Elderly

## Abstract

**Background:**

Clopidogrel is an antiplatelet drug used in the treatment of ischemic stroke. Safety and efficacy of clopidogrel has been confirmed in CAPRIE, PRoFESS trials. However, these studies focused on patients aged less than 75 years. CYP2C19 polymorphisms resulted in individual differences in clopidogrel response. Our objective was to determine whether elderly stroke patients aged over 75 years would benefit from CYP2C19-genotype-guided strategy for the secondary prevention of stroke.

**Methods:**

A retrospective analysis of patients aged 75 years or older with non-cardiogenic stroke who received 75 mg clopidogrel was performed. CYP2C19 genotype-guided group included noncarriers of CYP2C19*2 or CYP2C19*3 loss-of-function alleles (LoFA) and compared against the non-genotype-guided group which may carriers CYP2C19 LoFA or not. The primary endpoints were composite of stroke and myocardial infarction at 24 months’ follow-up.

**Results:**

Two hundred one patients were included: 99 in the genotype-guided group and 102 in the non-genotype-guided group. Kaplan-Meier(KM)analysis showed that CYP2C19 gene polymorphism was associated with the rate of the primary endpoints (*P* = 0.0031). The primary endpoints occurred in 13 patients (13.1%) in the genotype-guided group and in 30 patients (29.4%) in the non-genotype-guided group (hazard ratio(HR), 0.39; 95% confidence interval(CI), 0.20 to 0.75; *p* = 0.004). Cox regression analysis showed that CYP2C19 genotype-guided strategy was a protective factor for the primary endpoints (HR, 0.39; 95% CI:0.20 to 0.74, *P* = 0.004).

**Conclusion:**

The CYP2C19 genotype-guided strategy could reduce the occurrence of composite of stroke and myocardial infarction compared to a non-genotype-guided strategy for non-cardiogenic stroke patients aged 75 years or older who received clopidogrel.

## Background

At present, antiplatelet therapy is the cornerstone of ischemic stroke treatment. Clopidogrel and aspirin are common anti-platelet aggregation drugs and they are the first-line drugs for the secondary prevention of non-cardiogenic stroke. The efficacy and safety of a daily 75-mg dose of clopidogrel to prevent stroke recurrence has been extensively validated in large-scale randomised control trials (such as CAPRIE, PRoFESS), and it is also recommended by national guidelines [1, 2]. The ESSEN Stroke Risk Score (ESRS) is a scoring model of high-risk factors for ischemic stroke recurrence constructed according to the CAPRIE and REACH trials, which showed that high-risk patients (ESRS ≥3) treated with clopidogrel had better results than those treated with aspirin [1, 3]. However, in clinical practice, it was also found that although many patients took clopidogrel regularly, they did not achieve the expected anti-platelet aggregation effect, and they still experienced adverse vascular events such as stroke recurrence or myocardial infarction which is called clopidogrel resistance(CR). There are many factors including both genetic factors such as pharmacodynamics/pharmacokinetics-related genes, and non-genetic factors such as drug interactions, patients’ accompanying diseases, poor medication adherence, advanced age and so on which leading to CR [4, 5]. A large number of studies showed that polymorphisms of genes related to clopidogrel absorption, biological metabolism, and receptor binding were important factors of CR. The different genotypes led to individual differences in clopidogrel metabolism and efficacy, and ultimately resulted in completely different clinical outcomes, among which CYP2C19 gene polymorphism attracted much attention [4–8].

Clopidogrel is a prodrug that needs to be metabolized by the liver cytochrome P450 (CYP450) enzymes to be converted into an active drug. CYP2C19 is one of the genes that plays a major role in the CYP450 system, directly participating in the conversion of clopidogrel to its active metabolite. At present, there are more than 25 known types of CYP2C19 mutations, among which CYP2C19*2, CYP2C19*3 and CYP2C19*17 are the most common mutant alleles. The two mutant alleles of CYP2C19*2 and CYP2C19*3 are the main LoFA, which can lead to reduction or complete loss of CYP2C19 enzyme activity, so that clopidogrel metabolism is inhibited. CYP2C19*17 is a gain-of-function allele and enhances clopidogrel metabolism [5–8]. The subgroup analysis of CHANCE and subsequent meta-analysis showed that the presence of CYP2C19 LoFA was associated with the decreased efficacy of clopidogrel in the treatment of acute ischemic stroke or transient ischemic attack (TIA) [9, 10]. However, there were also some controversies. Some studies suggested that for carriers of CYP2C19 LoFA with ESRS ≥3, clopidogrel combined with aspirin for TIA or acute cerebral infarction could still significantly reduce 90-day stroke recurrence and the incidence of combined vascular events compared with aspirin alone [11].

At present, elderly patients aged over 75 years were often excluded from large clinical studies. The occurrence of ischemic stroke was obviously related to age: the older the patients, the higher the risk of ischemic stroke. Its prevalence in very elderly persons (≥80 years of age) is 13.8–14.9%, and the number of elderly patients accounts for 20% of all acute ischemic strokes [12]. At the same time, the elderly patients with ischemic stroke are characterized by high mortality and disability rate, and the clinical outcome was very poor. With the development of our country’s aging population, the ischemic stroke of the elderly population has received increasing attention. The purpose of this study therefore is to determine whether CYP2C19 genotype-guided strategy for selection of clopidogrel in elderly patients aged over 75 years with ischemic stroke can reduces the occurrence of end events and better prevent stroke recurrence?

## Methods

### Study population

This is a retrospective analysis of elderly patients with acute ischemic stroke who was prescribed clopidogrel during hospitalization and after being discharged from the Department of Neurology, Chaoyang Hospital and Shijingshan Hospital from May 2016 to May 2018. They were divided into two groups. Patients included in the non-genotype-guided group were recruited from May, 2016 to February, 2017 who took clopidogrel without CYP2C19 testing (102 cases), and may or may not carry CYP2C19 LoFA. Patients included in the genotype-guided group were recruited from March, 2017 to May, 2018 who took clopidogrel under the guidance of CYP2C19 (99 cases), and did not carry the CYP2C19 LoFA.

Inclusion criteria: 1. Acute cerebral infarction confirmed by head CT or head MRI; 2. Aged over 75 years; 3. Non-cardiogenic stroke; 4. NIHSS of less than 15 and mRS less than 3 in acute stage after cerebral ischemia; 5. long-term regular use of 75 mg clopidogrel during follow-up.

Exclusion criteria: 1. Malignant tumor; 2. Severe liver and kidney cardiopulmonary insufficiency (Severe liver insufficiency refers to transaminase exceeds normal 3 times above. Severe renal insufficiency refers to serum creatinine ≥177 μmol/L. Severe cardiac insufficiency refers to left ventricular ejection fraction ≤30%); 3. Taking aspirin or other antiplatelet aggregation and anticoagulant drugs during follow-up; 4. Hemorrhagic disease; 5. Autoimmune disease; 6. Platelet count less than 100 × 10^9^/L or more than 500 × 10^9^/L; 7. Hemorrhagic transformation after infarction.

This study was approved by the ethics committees at Chaoyang Hospital and Shijingshan Hospital.

### Genotyping

Three single-nucleotide polymorphisms (SNPs) for CYP2C19 including CYP2C19*2(681 G > A, rs4244285), CYP2C19*3(636 G > A, rs4986893)and CYP2C19*17(− 806 C > T, rs12248560)were tested by Digital Fluorescence Molecular Hybridization (DFMH) using a commercial kit from Sino Era Genotech (http://www.sino-era.com/, Beijing, China).

### Clinical outcomes

All patients were followed for a period of 2 years, by way of telephone calls, outpatient reviews, appointment interviews, and readmission records, to get to know the patient’s general condition, medication status, and the occurrence time of the endpoint. The primary endpoints referred to the composite of stroke (including ischemic or hemorrhagic) and myocardial infarction during the two-year follow up. The secondary endpoints referred to any first recurrent ischemic stroke, hemorrhagic stroke, myocardial infarction and all-cause mortality. Recurrent ischemic stroke was defined as symptoms of neurological deficit during follow-up, lasting more than 24 h, and head CT or MRI confirmed the corresponding ischemic lesions [13]. Cerebral hemorrhage referred to non-traumatic intraparenchymal hemorrhage [14]. Myocardial infarction was defined by the third global definition of acute myocardial infarction [15].

### Statistical analyses

The baseline characteristics were compared between two groups categorized by the use of CYP2C19 genotype-guided strategy. Quantitative data (e.g., age) were presented as medians (interquartile ranges) and the nonparametric Wilcoxon test was used to compare the two groups. Categorical variables (e.g., hypertension, diabetes and so on) were presented as percentages and tested by chi-square test or fisher’s accurate probability method according to the sample sizes of the groups. The Kaplan Meier curve described the survival rate of patients without clinical endpoints in the two groups, and Log rank was used to compare the difference between the two groups. A multivariate COX regression model was used to analyze the risk factors associated with clinical endpoints. Two-sided *P* < 0.05 was considered statistically significant. All statistical analyses were conducted with SPSS software version 22.0.

## Results

**Fig. 1:**
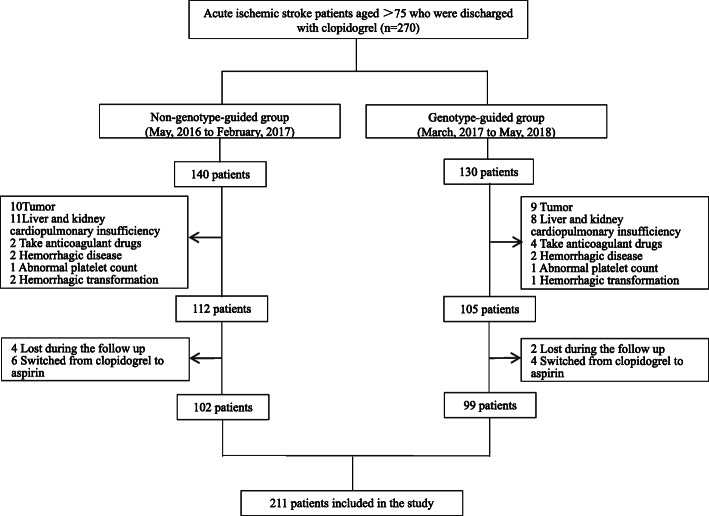
Study profile

**Fig. 2:**
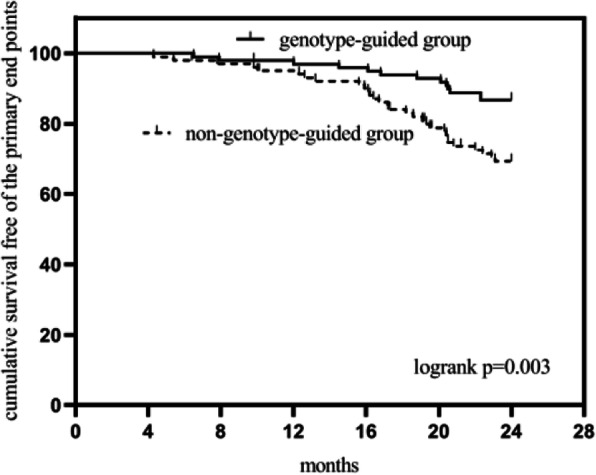
Cumulative Kaplan-Meier survival for the primary end points of two groups

This study initially included 270 patients aged over 75 years with non-cardiogenic non-disabling stroke who took 75 mg of clopidogrel daily. According to the exclusion criteria, 53 patients were excluded. During follow-up, 10 patients changed from clopidogrel to aspirin, and 6 were lost (Fig.1). Two hundred one patients were finally included in the data analysis, and they were divided into 2 groups according to whether they took clopidogrel under the guidance of CYP2C19. In the non-genotype guided group there were 102 patients, while in the genotype-guided group there were 99 patients. The basic characteristics of the two groups of patients suggested that there were no significant differences between the two groups of patients in terms of age, Sex, medical history (including hypertension, diabetes, myocardial infarction, other cardiovascular diseases, peripheral vascular disease, kidney disease, smoking and drinking, etc.), ESRS, administration of concomitant drugs (Table 1). After follow-up, the KM survival curves of non-primary endpoints in the genotype-guided group and the non-genotype-guided group are shown in Fig.2, and the difference between the groups was statistically significant by the Log rank test (*P* = 0.003).

**Table 1:** Baseline clinical characteristics of two groups

	Genotype-guided group (*n* = 99)	Non-genotype-guided group (*n* = 102)	*p*-Value
Sex (women), n (%)	38(38.3)	39(38.2)	0.98
Age in years, median (IQR)	80(78–83)	81(77–84)	0.91
Medical history, n (%)			
Ischemic stroke	34(34.3)	29(28.4)	0.37
Hypertension	66(66.6)	65(63.7)	0.66
Diabetes mellitus	34(34.3)	30(29.4)	0.45
Myocardial infarction	8(8.0)	6(5.8)	0.54
Other heart diseases^a^	28(28.2)	26(25.4)	0.66
Peripheralvascular diseases	1(1.0)	3(2.9)	0.64
Hyperlipemia	34(34.3)	33(32.3)	0.77
Nephropathy	5(5.0)	10(9.8)	0.20
Current smokers	4(4.0)	4(3.9)	1.00
Alcohol drinking	2(2.0)	4(3.9)	0.71
ESRS (≥3), n (%)	87(87.8)	91(89.2)	0.77
Concomitant medication, n (%)			
Antihypertensive agents (CCB)	36(36.3)	38(37.2)	0.90
Proton pump inhibitors	5(5.0)	4(3.9)	0.96
Lipid-lowering agents, n (%)			
Atorvastatin	46(46.4)	42(41.1)	0.45
Rosuvastatin	44(44.4)	49(48.0)	0.61
Pitavastatin	9(9.1)	11(10.8)	0.69

^a^Other cardiovascular disease included angina pectoris and congestive heart failure

During the follow-up period, the primary endpoints occurred in 13 cases (13.1%) in the genotype-guided group, and 30 cases (29.4%) in the non-genotype-guided group (HR, 0.39; 95% CI, 0.20 to 0.75; *p* = 0.004). As for secondary endpoints, recurrent stroke (including ischemic stroke and hemorrhagic stroke) occurred in 12 cases (12.1%) in the genotype-guided group, and 28 cases (27.5%) occurred in the non-genotype-guided group (HR, 0.39; 95% CI, 0.20 to 0.76; *p* = 0.006), of which, ischemic stroke occurred in 11 cases (11.1%) of ischemic stroke in the genotype-guided group and 26 cases (25.4%) in the non-genotype-guided group (HR, 0.38; 95% CI, 0.19 to 0.77; *p* = 0.007). There was no significant difference between the two groups in terms of the incidence of hemorrhagic stroke, myocardial infarction, and all-cause mortality (Table 2).

**Table 2:** The primary and secondary endpoints of two groups

Endpoints	Genotype-guided group (n = 99)	Non-genotype-guided group (n = 102)	HR(95%CI)	*p*-Value
The primary endpoints (a composite of stroke and myocardial infarction)	13(13.1%)	30(29.4%)	0.39(0.20–0.75)	0.004
The secondary end points				
Recurrent stroke	12(12.1%)	28(27.5%)	0.39(0.20–0.76)	0.006
Ischemic stroke	11(11.1%)	26(25.4%)	0.38(0.19–0.77)	0.007
Hemorrhagic stroke	1(1.0%)	2(2.0%)	0.44(0.04–4.88)	0.505
Myocardial infarction	1(1.0%)	2(2.0%)	0.45(0.04–4.92)	0.509
All-cause mortality	3(3.0%)	8(7.8%)	0.34(0.09–1.29)	0.114

In order to correct the influence of age, hypertension, diabetes, hyperlipidemia, smoking, history of previous stroke and other factors on the primary endpoints, a multivariate COX regression analysis was performed, and results showed that hypertension was an independent risk factor for the occurrence of the primary endpoint (HR, 2.60; 95%CI, 1.22 to 5.55; *P* = 0.014). With other factors unchanged, the risk of the primary endpoint in patients with hypertension was 2.598 times that of patients without hypertension. Smoking was also an independent risk factor for the primary endpoint (HR, 4.34; 95%CI, 1.44 to 13.11; *P* = 0.009). Moreover, genotype guidance was found to be a protective factor for the primary endpoint (HR, 0.39; 95% CI:0.20 to 0.74, *P* = 0.004) (Table 3).

**Table 3:** Multivariable Cox regression analysis of risk factors associated with the primary end points

Variables	wald	HR	95%CI	*p*-Value
Hypertension	6.09	2.60	1.22–5.55	0.014
Current smokers	6.79	4.34	1.44–13.11	0.009
Genotype-guide	8.18	0.39	0.20–0.74	0.004

## Discussion

This study focused on the elderly patients aged over 75 years. To our knowledge, there is no similar research report. This two-year follow-up study suggested that among elderly Chinese patients with non-cardiogenic stroke who received 75 mg of clopidogrel daily, there was significantly difference in non-primary endpoints between the genotype-guided group and the non-genotype-guided group. The incidence of the primary endpoint events (including ischemic stroke, hemorrhagic stroke, and myocardial infarction cases) of CYP2C19 genotype-guided group decreased compared to the non-genotype-guided group, and so did the recurrence rate of ischemic stroke. There were no significant differences in the incidence of secondary endpoint events including hemorrhagic stroke, myocardial infarction, and all-cause mortality. Multivariate COX analysis also suggested that the CYP2C19 genotype-guided treatment was a protective factor for the endpoint event. The reason is that clopidogrel itself is inactive, and CYP2C19 is one of the most important genes in the process of clopidogrel turning into active metabolites, and CYP2C19 polymorphisms are considered to be the most important factor for individual clopidogrel differences. Studies showed that after taking clopidogrel, patients with CYP2C19 LoFA had a low metabolic rate, weak antiplatelet effects, and high platelet activation and aggregation rates. Their clinical prognosis was poorer than those without CYP2C19 LoFA, and they were more susceptible to ischemic vascular adverse events [5–8, 16]. In light of this, the US FDA issued Black Box Warnings against clopidogrel three times within a year, suggesting that the association of CYP2C19 polymorphisms with clopidogrel metabolism and vascular adverse events was clear, should be paid attention to in the clinic practices and other kinds of antiplatelet drugs should be considered or the dose of clopidogrel should be increased to cope with the problem [17]. In this study, none of the patients in the genotype-guided group carried the CYP2C19 LoFA, and most patients in the non-genotype guided might carry the CYP2C19 LoFA, causing clopidogrel resistance, which furthermore affected their clinical outcomes.

In the field of cardiovascular disease, the correlation between clopidogrel treatment of patients with CYP2C19 LoFA and poor prognosis has been widely confirmed [5–7], and Claassens et al. found that the early use of P2Y12 inhibitors under the guidance of CYP2C19 genotype to treat patients who undergoing primary percutaneous coronary intervention could reduce thrombotic events and the risk of bleeding compared with standard treatment [18]. In the field of cerebrovascular, there are many studies suggesting the correlation between the two [9, 19, 20], but there is no research on the use of anti-platelet aggregation drugs under similar genetic guidance schemes. At present, a large-scale randomised, double-blind, controlled study in China is also underway: CHANCE-2 trial is also aimed at patients with acute cerebral infarction carrying the CYP2C19 LoFA. Under the genetic guidance, ticagrelor, another antiplatelet drug, is used instead of clopidogrel, and new and better drugs are expected to be found to prevent acute cerebral infarction. Jia et al. tested the CYP2C19 genotypes of 259 patients with acute cerebral infarction and evaluated the prognosis of stroke by mRS, and its results showed that the clinical outcomes of CYP2C19 LoFA carriers at 3 and 6 months after stroke were worse than non-carrying patients [20]. The genetic study of the CHANCE subgroup showed that in patients with acute ischemic stroke or transient ischemic attack treated with clopidogrel, those with the CYP2C19 LoFA were more at risk of stroke and composite vascular events than those without, and the presence of CYP2C19 LoFA reduced the efficacy of clopidogrel by 20% [9]. This result is similar to this study, which suggests that CYP2C19 genotype-guided treatment can reduce the recurrence rate of ischemic stroke, but it cannot reduce the risk of bleeding, which may be related to the small sample size of this study.

There are also some different voices. Korean scholars suggested that there were no significant differences between a poor CYP2C19 genotype for clopidogrel metabolism and a good CYP2C19 genotype in the rates of stroke recurrence, major vascular events for the secondary prevention of stroke [21]. Osnabrugge et al. claimed that the heterogeneity and publication bias of a large number of studies meant that the available evidence for personalized antiplatelet management based on the CYP2C19 genotype was insufficient [22]. Some domestic studies believed that the CYP2C19 genotype contributed only 12% to the heterogeneity of clopidogrel response, and there was still a large gap between the genotype and clinical outcomes. At the same time, it was proposed that in the CYP2C19 LoFA population, such as ESRS ≥3, clopidogrel still had a significant effect on acute ischemic stroke or transient ischemic attack [11]. The results of this study are inconsistent with the above studies, because the genotype distribution of CYP2C19 has obvious ethnic differences. The patients in this group are all Han Chinese, and the Chinese CYP2C19 LoFA carrying rate is as high as 58.8% [9], which is significantly higher than that of the Western population at 30% [23]. In addition, the research subjects in our study are elderly patients with many coexisting diseases. The ESRS scores of 88.6% of patients have greater than or equal to 3 points, of which age and other risk factors account for a certain proportion. In comparison, the conclusion by CHANCE studies that clopidogrel still has a significant effect for patients with ESRS≥3 focuses on high-risk stroke patients, whose risk factors account for more in the ESRS scores. In addition, the two groups are also different in age: the latter’s average age is only 62 years old.

This study also has some shortcomings. First, this study was a two-center retrospective study with a relatively small sample size and relatively short follow-up time. Secondly, all patients were only tested for CYP2C19*2 and*3, and it is still uncertain whether there are other candidate alleles involved, so there may be some deviations in the research results.

In conclusion, we discovered CYP2C19 polymorphisms were associated with the clinical outcome of elderly ischemic stroke patients by comparing whether they took clopidogrel under the guidance of CYP2C19. The incidence of endpoints (including ischemic stroke, hemorrhagic stroke, and myocardial infarction) in patients taking clopidogrel under the guidance of the CYP2C19 was low. Therefore, clinical testing of CYP2C19 genotype can be used to assess the incidence of clopidogrel resistance risks, which can inform the treatment plan of clinical anti-platelet aggregation for the secondary prevention of stroke.

## Data Availability

The data in this study are available from the corresponding author upon reasonable request.

## References

[1] CAPRIE Steering Committee (1996). A randomised, blinded, trial of clopidogrel versus aspirin in patients at risk of ischaemic events (CAPRIE). CAPRIE steering committee. Lancet.

[2] Sacco RL, Diener HC, Yusuf S, Cotton D, Ounpuu S, Lawton WA (2008). Aspirin and extended-release dipyridamole versus clopidogrel for recurrent stroke. N Engl J Med.

[3] Weimar C, Diener HC, Alberts MJ, Steg PG, Bhatt DL, Wilson PW (2009). The Essen stroke risk score predicts recurrent cardiovascular events: a validation within the reduction of Atherothrombosis for continued health (REACH) registry. Stroke..

[4] Wiviott SD, Antman EM (2004). Clopidogrel resistance: a new chapter in a fast-moving story. Circulation..

[5] Pereira NL, Rihal CS, So DYF, Rosenberg Y, Lennon RJ, Mathew V (2019). Clopidogrel Pharmacogenetics. Circ Cardiovasc Interv.

[6] Mega JL, Close SL, Wiviott SD, Shen L, Hockett RD, Brandt JT (2009). Cytochrome p-450 polymorphisms and response to clopidogrel. N Engl J Med.

[7] Simon T, Verstuyft C, Mary-Krause M, Quteineh L, Drouet E, Méneveau N (2009). Genetic determinants of response to clopidogrel and cardiovascular events. N Engl J Med.

[8] Klein MD, Lee CR, Stouffer GA (2018). Clinical outcomes of CYP2C19 genotype-guided antiplatelet therapy: existing evidence and future directions. Pharmacogenomics..

[9] Wang Y, Zhao X, Lin J, Li H, Johnston SC, Lin Y (2016). Association between CYP2C19 loss-of-function allele status and efficacy of Clopidogrel for risk reduction among patients with minor stroke or transient ischemic attack. JAMA..

[10] Pan Y, Chen W, Xu Y, Yi X, Han Y, Yang Q (2017). Genetic polymorphisms and Clopidogrel efficacy for acute ischemic stroke or transient ischemic attack: a systematic review and meta-analysis. Circulation.

[11] Xu J, Wang A, Wangqin R, Mo J, Chen Z, Dai L (2019). Efficacy of clopidogrel for stroke depends on CYP2C19 genotype and risk profile. Ann Neurol.

[12] Benjamin EJ, Virani SS, Callaway CW, Chamberlain AM, Chang AR, Cheng S (2018). Heart disease and stroke statistics-2018 update: a report from the American heart association. Circulation.

[13] Khanevski AN, Bjerkreim AT, Novotny V, Naess H, Thomassen L, Logallo N (2019). Recurrent ischemic stroke: incidence, predictors, and impact on mortality. Acta Neurol Scand.

[14] Domingues R, Rossi C, Cordonnier C (2015). Diagnostic evaluation for nontraumatic intracerebral hemorrhage. Neurol Clin.

[15] Thygesen K, Alpert JS, Jaffe AS, Simoons ML, Chaitman BR, White HD (2012). Third universal definition of myocardial infarction. J Am Coll Cardiol.

[16] Mega JL, Close SL, Wiviott SD, Shen L, Walker JR, Simon T (2010). Genetic variants in ABCB1 and CYP2C19 and cardiovascular outcomes after treatment with clopidogrel and prasugrel in the TRITON-TIMI 38 trial: a pharmacogenetic analysis. Lancet..

[17] Holmes DR, Dehmer GJ, Kaul S, Leifer D, O’Gara PT, Stein CM (2010). ACCF/AHA clopidogrel clinical alert: approaches to the FDA “boxed warning”: a report of the American College of Cardiology Foundation task force on clinical expert consensus documents and the American Heart Association endorsed by the Society for Cardiovascular Angiography and Interventions and the Society of Thoracic Surgeons. J Am Coll Cardiol.

[18] Claassens DMF, Vos GJA, Bergmeijer TO, Hermanides RS, van’t Hof AWJ, van der Harst P (2019). A genotype-guided strategy for Oral P2Y12 inhibitors in primary PCI. N Engl J Med.

[19] Han Y, Lv HH, Liu X, Dong Q, Yang XL, Li SX (2015). Influence of genetic polymorphisms on Clopidogrel response and clinical outcomes in patients with acute ischemic stroke CYP2C19 genotype on Clopidogrel response. CNS Neurosci Ther.

[20] Jia DM, Chen ZB, Zhang MJ, Yang WJ, Jin JL, Xia YQ (2013). CYP2C19 polymorphisms and antiplatelet effects of clopidogrel in acute ischemic stroke in China. Stroke..

[21] Han SW, Kim YJ, Ahn SH, Seo WK, Yu S, Oh SH (2017). Effects of Triflusal and Clopidogrel on the secondary prevention of stroke based on cytochrome P450 2C19 genotyping. J Stroke.

[22] Osnabrugge RL, Head SJ, Zijlstra F, ten Berg JM, Hunink MG, Kappetein AP (2015). A systematic review and critical assessment of 11 discordant meta-analyses on reduced-function CYP2C19 genotype and risk of adverse clinical outcomes in clopidogrel users. Genet Med.

[23] Levine GN, Jeong YH, Goto S, Anderson JL, Huo Y, Mega JL (2014). Expert consensus document: world heart federation expert consensus statement on antiplatelet therapy in east Asian patients with ACS or undergoing PCI. Nat Rev Cardiol.

